# An Interesting Case of a Bilious Pleural Effusion

**DOI:** 10.1177/2324709617720160

**Published:** 2017-07-21

**Authors:** Christoffel van Niekerk, Kelly Fan, Anna Sarcon, Bao Luu

**Affiliations:** 1 Scripps Green Hospital, La Jolla, CA, USA

**Keywords:** Cholethorax, bilothorax, ERCP, Complication

## Abstract

Malignant pleural effusions are common complications in patients with primary or metastatic cancer to the lungs. In this article, we describe a unique case of a patient with history of diffuse pulmonary metastases from gallbladder adenocarcinoma who acutely developed a bilious pleural effusion following endoscopic retrograde cholangiopancreatography. We believe the bilious pleural effusion (cholethorax or bilothorax) developed as a complication of endoscopic retrograde cholangiopancreatography rather than tumor burden causing a fistula from the biliary tree to the right pleural space. We discuss possible mechanisms of formation of the bilious pleural effusion in our patient and present a literature review of previously reported cases of bilious pleural effusions.

## Case Presentation

**Figure 1.**
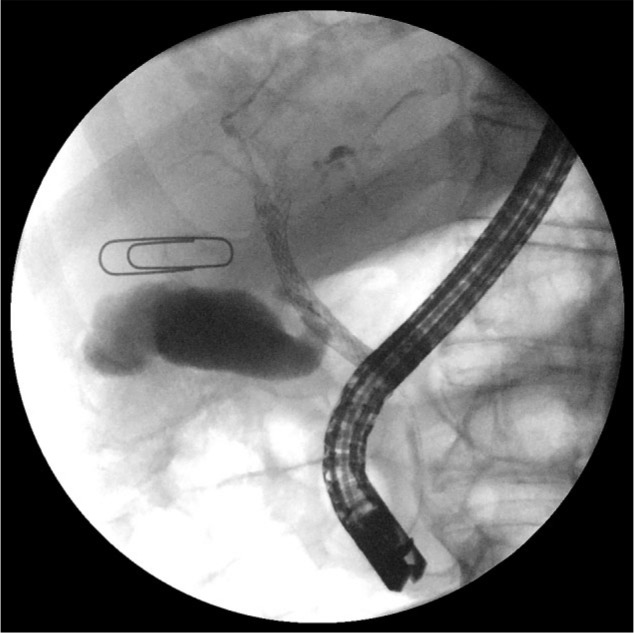
Fluoroscopic evidence of patent new biliary bare metal stent.

A 76-year-old man presented with mild abdominal pain, nausea without emesis, and new development of jaundice and dark urine. His medical history was significant for metastatic gallbladder adenocarcinoma diagnosed 6 months prior to this hospitalization by retroperitoneal lymph node biopsy following a positron emission tomography scan. He was treated with gemcitabine and oxaliplatin for 4 cycles, followed by folinic acid, fluorouracil, irinotecan, and bevacizumab. He also had 1 cycle of erlotinib prior to admission. Exam revealed a jaundiced and cachectic man. He was afebrile, with stable vital signs. Abdominal exam revealed mild tenderness to palpation in the right upper quadrant without any rebound or guarding. Laboratory values were significant for aspartate aminotransferase (AST) 544, alanine aminotransferase (ALT) 1272, total bilirubin 6.7, and alkaline phosphatase 697 with normal baselines days prior. Computed tomography (CT) scan of the abdomen and pelvis with contrast showed severe intrahepatic ductal dilation without common bile duct obstruction. Available images at the bases of the lungs did not reveal a pleural effusion; however, previously noted diffuse metastatic disease in his lungs was once again appreciated. He was diagnosed with obstructive jaundice and underwent endoscopic retrograde cholangiopancreatography (ERCP) with biliary sphincterotomy and subhilar 4 cm ×y 10 mm metal stent placement with confirmed biliary drainage on fluoroscopy (Figure 1). He was continued on ciprofloxacin. At the time of discharge, his laboratory values improved with AST 303, ALT 770, total bilirubin 3.7, and alkaline phosphatase 676.

**Figure 2.**
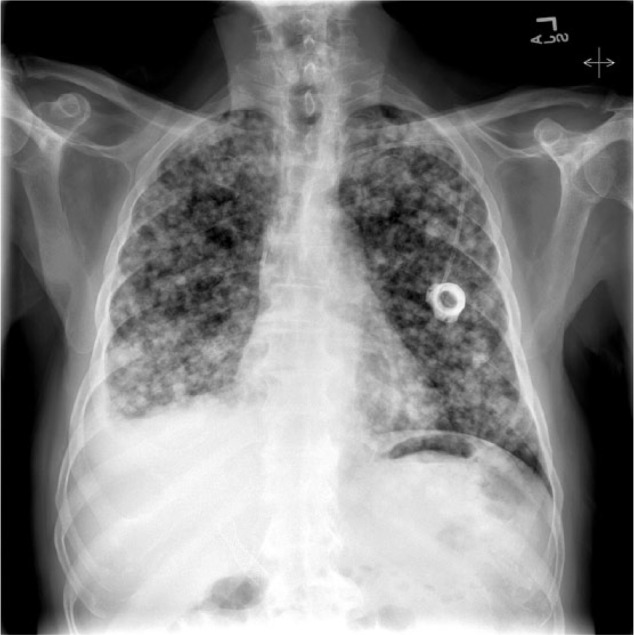
Right-sided pleural effusion and pulmonary and pleural metastases.

**Figures 3 and 4.**
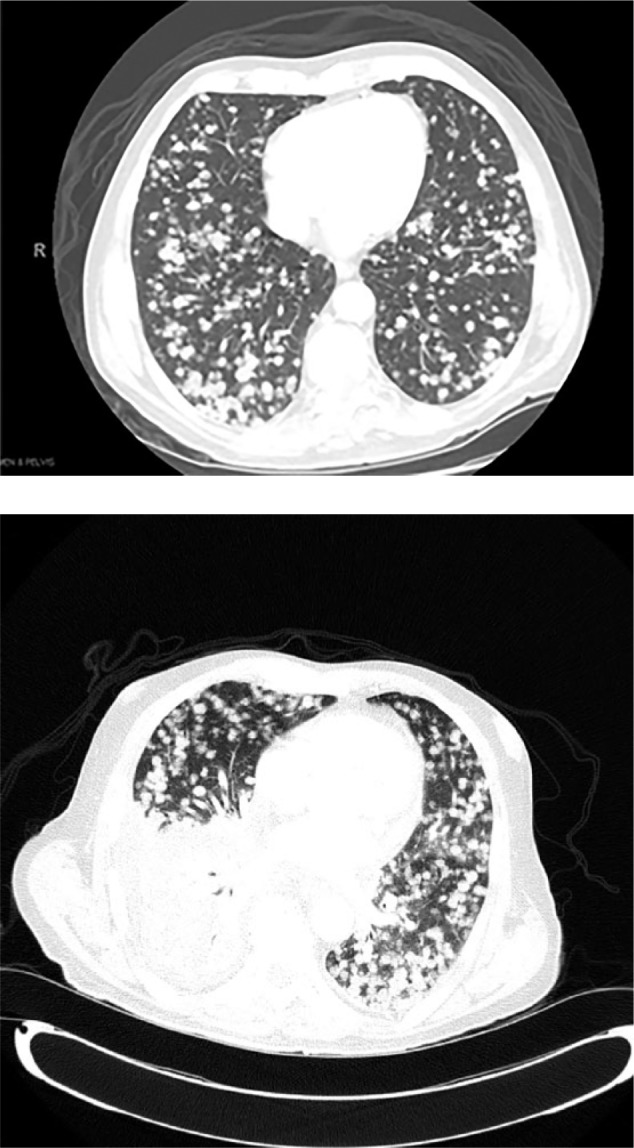
Before and after CT scans of the chest with contrast showing pulmonary metastasis and newly.

**Figure 5.**
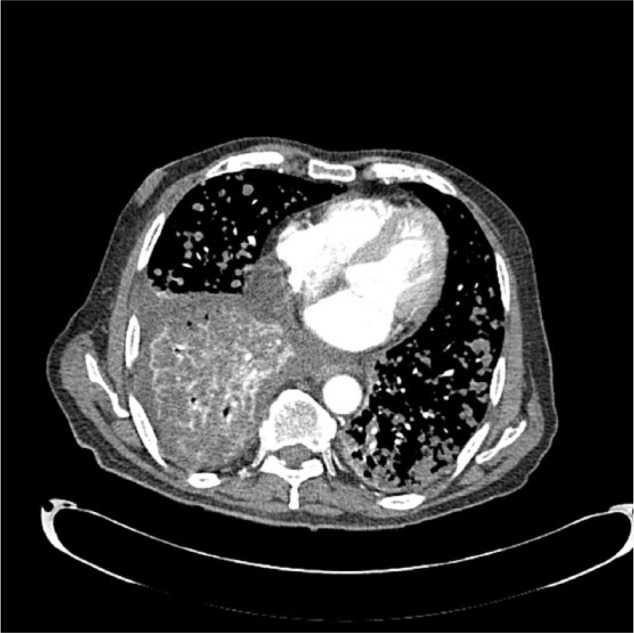
Mediastinal window showing multiple pulmonary nodules and large right pleural effusion.

**Figure 6.**
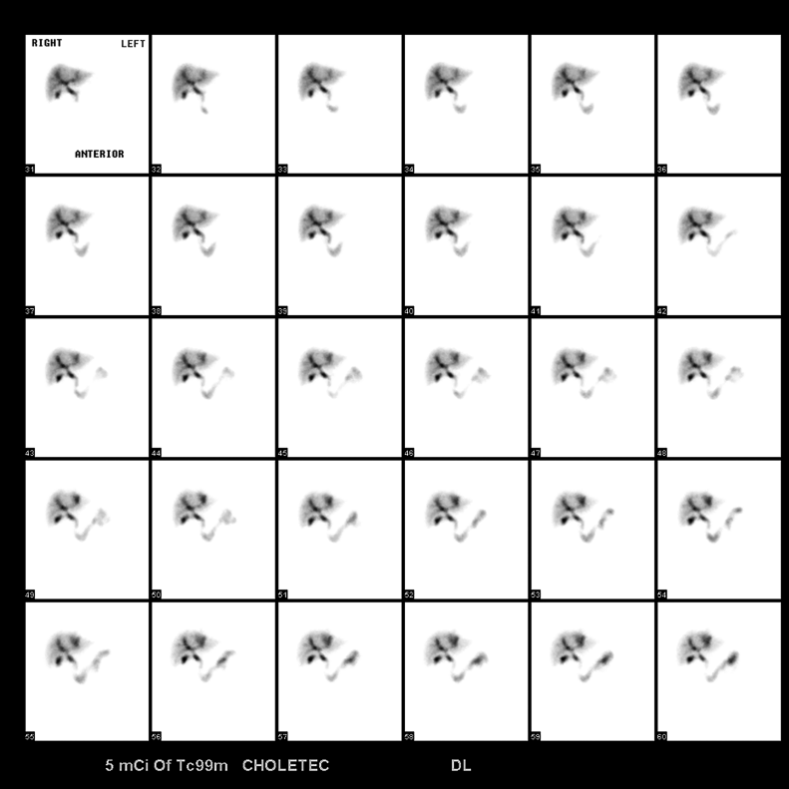
HIDA scan showing biliary leak over the left lobe of the liver.

He was readmitted 5 days after his ERCP with rigors and chills. Physical exam was significant for new development of decreased breath sounds in the right lower lung fields and mild persistent abdominal pain in the right upper quadrant. His laboratory values were significant for a leukocytosis of 21.7 with 87.6% neutrophils, AST 115, ALT 426, total bilirubin 3.1, and alkaline phosphatase 572. Chest X-ray and CT chest showed interval development of a right-sided pleural effusion with patency of the proximal subsegmental bronchi leading to the right lower lobe (Figures 2-4). His antibiotics were broadened to vancomycin and piperacillin/tazobactam for presumed aspiration pneumonia versus cholangitis/cholecystitis. A thoracentesis was performed, which revealed a green pleural effusion. Laboratory results were suggestive of an exudative pleural effusion (Figure 5) with lactate dehydrogenase 1246. Bilirubin in the fluid was elevated at 33.9, cytology was negative for malignant cells, and pleural effusion cultures remained negative. HIDA scan showed a biliary leak over the left lobe of the liver (Figure 6). Diagnosis of bilious pleural effusion (cholethorax or bilothorax) was made. Ultimately, he declined further therapeutic options; however, his respiratory symptoms remained stable at the time of discharge on nasal cannula oxygen. Given the poor prognosis of his underlying metastatic gallbladder cancer, he elected to receive hospice care.

## Discussion

Gallbladder cancer (GBC) is a rare and aggressive cancer with an estimated incidence rate of 1 to 2 per 100 000. Stage IV 5-year survival rate of gallbladder cancer is 2%.^1^ The reason for such a poor prognosis is the often late disease presentation. Once suspected by ultrasonic evidence of intracholecystic mass, a CT or magnetic resonance imaging of the abdomen should be pursued followed by a biopsy for pathologic confirmation. Surgery is the only potential cure for early staged GBC but palliative measures are the primary options in metastatic cases. Specifically for our patient, biliary obstruction can often be alleviated by ERCP with biliary decompression with stent placement or percutaneous drain placement. Complication rates for ERCP with stent placement are low with 3.5% risk of developing pancreatitis, 1.3% risk of hemorrhage, 1% risk of cholangitis, and only 0.6% risk of perforation.^2^

Malignant pleural effusions are a common presentation in patients with metastatic disease to the pleura and lung parenchyma. Development of pleural effusions is often subacute in timing and significantly decreases quality of life. Treatment options include indwelling pleural catheters versus pleurodesis.^3^ Our case is unique in the rapid development of the pleural effusion. Our patient had documented CT imaging studies without evidence of pleural effusion just 5 days prior to onset of his pleural effusion. Furthermore, he had laboratory studies confirming the presence of bilirubin in his pleural fluid. HIDA scan also showed evidence of leakage of bilious content into the left lobe of the liver. Taken together, an iatrogenic bilious pleural effusion secondary to a complication from his ERCP with metal stent placement seems most likely. While there is a possibility of spontaneous fistula, the timing of the leak shortly after the ERCP and rapidity of the large effusion makes a spontaneous fistula much less likely.

In our literature review of bilious pleural effusions (cholethorax or bilothorax), we note that this is a rare presentation often resulting from a procedural complication involving the gallbladder and biliary tree.^4-8^ An early reported case of a bilious pleural effusion occurred in a patient with obstructive jaundice who underwent percutaneous catheter biliary decompression.^6^ Subsequent cases have described bilious pleural effusions following a liver biopsy and ERCP with stone removal in a liver transplant patient,^7^ complications from presumed intrahepatic biliary stent migration,^5^ and complication from a laparoscopic cholecystectomy.^4^ The presumed mechanism of bilious pleural effusion formation in each of these cases is small perforations in the diaphragm leading to fenestration of contents from the peritoneal cavity into the pleural space.^4-8^ Our patient likely had a similar mechanism of injury in which a biliary tract perforation from ERCP or stent migration lead to bilious content leakage into the left lobe of the liver. Subsequently, the accumulated bilious fluid migrated through the diaphragm and caused the bilious pleural effusion, similar to the mechanism in the formation of a hepatic hydrothorax. There was no noted diaphragmatic injury during the ERCP in our patient. While we were able to establish the diagnosis of a bilious pleural effusion with thoracentesis, an autopsy would be needed to definitively pinpoint a mechanism of formation.

In summary, despite low complication rates, ERCP with stent placement is not a benign procedure and does have a 0.6% risk of biliary ductal perforation, which likely occurred in our patient. Case reports on bilious pleural effusions suggest low risk of persistent pleural fluid re-accumulation following thoracentesis.
